# Bacterially Converted Oat Active Ingredients Enhances Antioxidative and Anti-UVB Photoaging Activities

**DOI:** 10.1155/2022/1901564

**Published:** 2022-05-28

**Authors:** Jennifer K. Lee, Inyong Kim, Eun-Kyeong Jeon, Jung-Heun Ha, Cher-Won Hwang, Jin-Chul Kim, Woong-Suk Yang, Hyunju Choi, Hee-Do Kim, Cheorl-Ho Kim

**Affiliations:** ^1^Food Science & Human Nutrition Department, University of Florida, Gainesville 32611, FL, USA; ^2^Department of Food and Nutrition, Sunchon National University, Sunchon, Republic of Korea; ^3^School of Life Science, Handong Global University, Pohang 37554, Gyeongsangbuk-do, Republic of Korea; ^4^Department of Food Science and Nutrition, Dankook University, Cheonan 31116, Chungcheongnam-do, Republic of Korea; ^5^Global Leadership School (GLS), Handong Global University, Pohang 37554, Gyeongsangbuk-do, Republic of Korea; ^6^Natural Product Research Institute, Korea Institute of Science and Technology (KIST), Gangneung 25451, Gangwon-do, Republic of Korea; ^7^Nodaji Co., Ltd, Pohang 37927, Gyeongsangbuk-do, Republic of Korea; ^8^Department of Biological Sciences, SungKyunKwan University, Suwon 16419, Gyungki-do, Republic of Korea

## Abstract

Oat (*Avena sativa* L.) is one of the most widely consumed cereal grains worldwide and is considered as an important cereal crop with high nutritional value and potential health benefits. With different bacterial strains, fermented oat extracts were examined for the antioxidant and antiaging effects on the skin after optimization of extraction conditions. Fermented oats contained high avenanthramides, and its function was investigated on matrix metalloproteinase-1 and collagen expression with human dermal fibroblast cells. After fractionation, butanol layers showed the highest avenanthramides contents. Therefore, the microbial fermentation of oats enhances the quality and content of functional ingredients of oats, which provide natural dietary supplements, antioxidants, and antiaging agents.

## 1. Introduction

Skin aging is caused by several factors, mainly genetic and environmental stressors. Among numerous factors involved in skin aging, UV-induced damage is typically referred to as photoaging [1]. In particular, UVB radiation is the main reason for skin alteration such as erythema, hyperpigmentation, photoaging, immune suppression, and cancer due to stimulation of skin cells [2, 3]. UVB exposure generates reactive oxygen species (ROS) in skin cells and activates aging-related signal transduction system by promoting oxidative stress, leading to decreased synthesis of extracellular matrix proteins such as collagen and elastin in connective tissues of the skin by binding to unsaturated fatty acids and proteins [4]. Collagen, a major matrix protein synthesized in fibroblasts and degraded by matrix metalloproteinase (MMPs), accounts for 70–80% of the dry weight of skin tissue. It plays an important role in providing strength, maintaining tension, and protecting the skin from external stimuli [5]. MMPs are secreted from keratinocytes and fibroblasts of the skin. They are classified as a group of proteolytic enzymes such as collagenase (MMP-1 and MMP-13), gelatinase (MMP-2 and MMP-9), stromelysin (MMP-3), and matrilysin (MMP-7) that mainly degrade the extracellular matrix and basement membrane [6].

Oat (*Avena sativa* L.) is one of the most widely consumed cereal grains worldwide. It is mainly cultivated in temperate regions in American and European countries, including Russia, Canada, and the United States [7]. Oat is considered an important cereal crop as it possesses high nutritional value and potential health benefits associated with dietary fibers, particularly *β*-glucan and starch, globulin, unsaturated fatty acids, and phytochemicals [8]. Studies have well demonstrated that high *β*-glucan content in oats is associated with a reduced risk of coronary heart disease (CHD) by lowering blood cholesterol levels [9, 10] and intestinal absorption of glucose. In addition, oats possess antioxidant components such as vitamin E, phenolic compounds, phytic acid, flavonoids, and avenanthramide [11, 12].

Avenanthramides (AVAs) are low molecular weight, soluble phenolic compounds mainly concentrated in oat leaves and seeds. AVAs exhibit antioxidant, anti-inflammatory, anticancer, and anti-proliferative effects [13, 14]. Moreover, AVAs can act as phytoalexins in response to pathogen infection (e.g., fungal growth) or elicitor treatments [15, 16]. Antioxidant activities of AVAs have been reported to be 10–30 times greater than those of other phenolic compounds in oats. Of 25 different types of AVAs identified to date, three isoforms, 5-hydroxyanthranilic acid with *p*-coumaric (AVA-A), ferulic (AVA-B), and caffeic (AVA-C) acids [17, 18], are mainly found in higher concentrations than other isoforms. Among AVAs, AVA-C accounts for about one-third of total AVA concentration in oat grain and exhibits the highest antioxidant *in vitro* [19].

Fermentation is one of the oldest food processing technologies to convert food components with controlled microbial reactions through enzymatic activities [20]. Food fermentation processes lead to enhanced nutritional value, taste, stability, and biochemical quality by involving different microorganisms [21, 22]. Studies have revealed that fermented foods can promote health by lowering blood cholesterol levels, protecting against pathogens and carcinogenesis, and alleviating symptoms related to osteoporosis, diabetes, obesity, and allergies [23, 24].

In this study, the benefit of oat extracts fermentation was investigated on antiaging of the skin and the antioxidative effect of different fermentation processes.

## 2. Materials and Methods

### 2.1. Materials and Extraction Conditions

The naked oat cultivar Choyang has been developed by Nodaji Co., Ltd., through organic farming. Oat grains used in this study were harvested in 2020 and obtained from Pohang-si, Gyeongsangbuk-do, Korea. Oat powder samples were mixed with 100 mL of 0, 20, 40, 60, 70, 80, and 100% (v/v) EtOH. To find the optimal EtOH ratio for extraction, 2,2-diphenyl-1-picrylhydrazyl (DPPH) radical scavenging activity, matrix metalloproteinase-1 (MMP-1)-mediated collagen degradation, and collagen expression were determined.

### 2.2. DPPH Radical Scavenging Activity

Free radical scavenging activity of oat was measured by DPPH assay as previously reported by Sharma et al. [25]. Briefly, 1.6 mL of 0.4 mM DPPH solution was added to 0.4 mL of oat sample extract followed by incubation at room temperature for 10 min. The absorbance was then measured at 525 nm. Ascorbic acid was used as the standard. Radical scavenging activity was expressed as a percentage of DPPH discoloration and calculated as follows:

(1) $$\begin{array}{c} \text{DPPH radical scavenging activity}\left(\%\right)=\left[\frac{\left(A0-A1\right)}{A0}\right]\times 100, \end{array}$$

where A_0_ was the absorbance of the DPPH solution, and A_1_ was the absorbance of the sample. The effective concentration (EC_50_) value was the concentration required to decrease the initial DPPH radical concentration by 50%.

### 2.3. Cell Culture Experiments

Human dermal fibroblasts (HDF; 1 × 10^5^ cells/mL) were cultured in DMEM medium (Hyclone, Logan, UT, USA) supplemented with 10% fetal bovine serum (FBS) and 100 U/ml penicillin/streptomycin at 37°C in a 5% CO_2_ incubator. Subsequently, subconfluent cells were subjected to treatment for 24 hours.

Testing the antiaging effect on the skin, cells were exposed to ultraviolet B (UVB) rays at a dose of 60 mJ/cm^2^ using a UVB source (Bio-Link Crosslinker, Vilber Lourmat, Cedex, France) set at a spectral peak of 312 nm for 20 seconds. After UVB irradiation, cells were cultured in a serum-free medium for 24 hours.

### 2.4. ELISA Assay

MMP-1 expression in HDF cells was measured using an MMP-1 DuoSet ELISA kit (R&D Systems, Minneapolis, MN, USA). Briefly, HDF cells were seeded into a 35 × 15 mm culture dish at a density of 1 × 10^5^ cells/mL and incubated at 37°C with 5% CO_2_. The next day, UVB alone or UVB/sample was treated for 24 hours, and then, the supernatant was analyzed. MMP-1 expression was calculated according to the calibration curve.

Type I procollagen level in HDF cells was measured using a procollagen I alpha 1 DuoSet ELISA kit (R&D Systems, Minneapolis, MN, USA). Briefly, HDF cells were seeded into a 35 × 15 mm dish at a density of 1 × 10^5^ cells/mL and incubated at 37°C with 5% CO_2_. The next day, the UVB alone or UVB/oat sample was treated for 24 hours, and then, the supernatant was collected for further experiments. The amount of each procollagen was calculated according to the calibration curve.

### 2.5. Fractionation of Extract

Considering DPPH radical scavenging activity, collagen expression, and MMP-1 inhibitory effect simultaneously, 20% ethanol extract was selected and subjected to fractionation. Oat powder samples were extracted with 20% ethanol at a solid to solvent ratio of 1 : 10 (w/v) at 70°C with shaking at 160 rpm for 24 hours followed by filtration through a Whatman No. 2 filter paper. Crude ethanol extracts were obtained using a rotary evaporator (N-1000, EYELA, Japan) followed by freeze-drying (PVTFD20RS, Ilshin Lab. Co. Ltd., Korea). This crude extract powder was stored at −80°C until further use.

### 2.6. Activities of Extracts Partitioned with Organic Solvents

**Figure 1:**
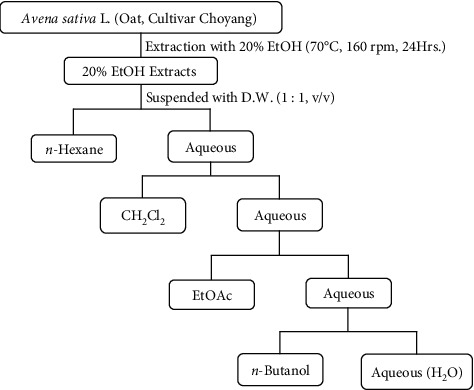
Procedures used for partitioning *Avena sativa* L.extract with organic solvents. HX, n-hexane; CH_2_Cl_2_, dichloromethane (MC); EtOAc, ethyl acetate; BuOH, *n-*butanol; H_2_O, residue from fractionation.

To investigate its constituents, 20% ethanol extract of oat (*Avena sativa* L.) was partitioned with *n*-hexane (Hx), dichloromethane (CH_2_Cl_2_, MC), ethyl acetate (EtOAc), *n*-butanol (BuOH), and residue solvent (H_2_O). In the case of fractionation of a solution, the solution extracted with 20% ethanol and *n*-hexane was mixed and left until layers were separated. The solvent was then recovered. The remaining solution was sequentially mixed with the extraction solution in the same manner to separate the dissolved material (Figure 1). The yield of ethanol extract was Hx, 0.9%; MC, 1.0%; EtOAc, 5.2%; BuOH, 22.9%; and water, 60.5%. Antioxidant activity, MMP-1 expression, and collagen expression in skin cells were then evaluated for these fractions.

### 2.7. Fermentation Conditions

Candidate strains for oat fermentation were as follows: *Bacillus subtilis* NDJ-002 (KCCM12379P, BS002), *Lactobacillus plantarum* YS-100 (KCCM12615P, LP100), and *Klyuveromyces marixanus* YS-091 (KCCM12635P, KM091). Fermented with 20% of ethanol (EtOH) extracts using these three strains were designated as BS002E, LPE, and KME, respectively. Ethanol (20%) extracts of raw oat (RO) and autoclaved (sterilized) oat (AO) powders were called ROE and AOE, respectively. To determine the antioxidant activities of these extracts, DPPH radical scavenging activity assay was performed. Ascorbic acid was used as a positive control.

### 2.8. Phenolic Contents

Total phenolic contents were measured according to the method described by Singleton [26] with minor modifications. Briefly, oat extract samples were diluted with distilled water to a final concentration of 10 mg/mL. They were then added with 0.2 mL of Folin-Ciocalteu's phenol reagent (Sigma Aldrich, Darmstadt, Germany) followed by incubation at room temperature for 3 minutes. Subsequently, 0.4 mL of a saturated Na_2_CO_3_ solution and 1.4 mL of distilled water were added to the reaction mixture followed by incubation at room temperature for 30 hours. Absorbance readings were taken at 760 nm. Tannic acid (Sigma-Aldrich, Darmstadt, Germany) was used as a reference standard. Total phenolic content was expressed as mg tannic acid equivalent (TAE)/g dry weight of plant powder.

### 2.9. Superoxide Dismutase (SOD)-Like Activity

The SOD-like activity was analyzed using an EZ-SOD assay kit (Dogen Bio Co. Ltd., Seoul, Korea). Briefly, 200 *μ*L of WST working solution and 20 *μ*L of Enzyme working solution were added to 20 *μ*L of samples. The mixture was reacted at 37°C for 20 minutes. Absorbance was then measured at 450 nm using a spectrophotometer.

### 2.10. Hydroxyl Radical Scavenging Activity

Hydroxyl radical activity with fermented oat extract was tested for antioxidant effect measurement. 10 mg/mL of extract was added to the reaction mixture in a final volume of 1 mL in potassium phosphate buffer (10 mM, pH 7.4). This mixture was incubated at 37°C for 2 h and then mixed with 1 mL of 2.8% TCA (w/v in water) and 1 mL of 1% thiobarbituric acid (TBA) (w/v). The mixture was heated in a boiling water bath for 15 min, and absorbance was taken at 532 nm. Thiourea was used as a positive control [27]. The hydroxyl radical scavenging activity (%) was calculated as follows:

(2) $$\begin{array}{c} \text{Hydroxyl radical scavenging activity}\left(\%\right)=\left[1-\left(\frac{\text{Asample}}{Ablank}\right)\right]\times 100, \end{array}$$

where *A*_sample_ is the absorbance of the sample, and *A*_blank_ is the absorbance of distilled water.

### 2.11. Gas Chromatography-Mass Spectrometry (GC/MS) Analysis

In order to assess changes in phytochemical compounds of fermented oats extracted with 20% ethanol, quantitative GC/MS was performed. The sample was dissolved in methanol at a final concentration of 10 mg/mL and then used for analysis. The sample was analyzed by a gas chromatography-mass spectrometry (GC/MS) system using an Agilent 7890B network GC system equipped with a 5977B network mass selective detector and an MPS2 series automated liquid sampler (Agilent Technologies Inc., Wilmington, DE, USA). The entire system was controlled by a Chemstation^®^ software (Agilent Technologies Inc.). A DB-5MS fused-silica capillary column (0.25 mm × 30 mm, 0.25 *μ*m) was used at the following conditions: temperature program of 70°C (1 min) to 280°C ramped at 5°C/min; carrier gas of heat a flow rate of 1 mL/min; a split ratio of 20 : 1; injection volume of 3 *μ*L for each sample; injection mode of splitless; and ion source type of EI.

### 2.12. High-Performance LC/MS Analysis

To determine the contents of avenanthramide and precursor (p-coumaric acid, ferulic acid, and caffeic acid) in fermented oats extracted with 20% ethanol, quantitative LC/MS was performed. Each sample was dissolved in distilled water or methanol at a final concentration of 10 mg/mL for analysis. Avenanthramide was quantified using the standard curve of each substance (avenanthramide A, avenanthramide B, and avenanthramide C). The precursor was quantified using the standard curve of each substance (*p*-coumaric acid, ferulic acid, and caffeic acid). LC/MS column was ACQUITY UPLC BEH C18 column, 2.7 mm × 50 mm, 1.8 *μ*m (Waters Milford, MA, USA). Conditions used for the analysis of LC/MS are described in Table 1.

### 2.13. Statistical Analysis

All results are expressed as mean ± standard deviation (SD). Data were analyzed by one-way ANOVA followed by Tukey's post hoc test. *P* < 0.05 was considered statistically significant. All statistical analyses were performed using the GraphPad Prism 8.0 version (GraphPad Software Inc., San Diego, CA, USA) based on the distribution of data.

## 3. Results and Discussion

### 3.1. Determination of Optimal Extraction Conditions

To determine the optimal ethanol ratio for oat extraction (Table 2), 70% and 100% EtOH fractions were the highest and lowest yield of 23.2% and 1.3%, respectively. Each fraction was tested for DPPH radical activity (EC_50_). Considering both yields and DPPH radical scavenging activity, 80% EtOH extraction condition was the most suitable for optimal extraction. However, considering the industrial application of oat products, under certain EtOH contents (below 50%) are required by law. Therefore, 20% of EtOH extracts were selected for further experiments.

### 3.2. Antiaging Effect of Skin with MMP-1 and Collagen Expression

**Figure 2:**
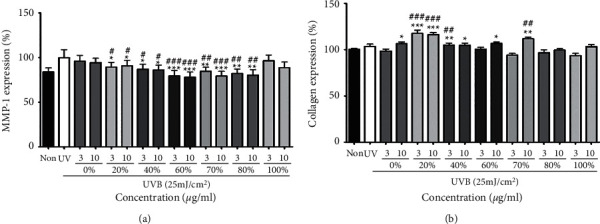
MMP-1 (a) and collagen (b) expression levels in groups treated with oats extracted with different ethanol concentrations. Each bar represents the mean ± SD of three replicates. ^*∗*^*p* < 0.05, ^*∗∗*^*p* < 0.01, ^∗∗∗^*p* < 0.001 versus UV-only group, ^#^*p* < 0.05, ^##^*p* < 0.01, ^###^*p* < 0.001 versus 0% EtOH extracts group. Data were analyzed statistically using one-way ANOVA followed by Tukey's post hoc test. Antiageing effect on dermal skin of fractionated oat extracts.

UV irradiation can lead to upregulation of MMP-1, which is an indicator of collagen degradation and skin damage. Inhibiting MMP-1 activation can reduce damaged skin cells [28]. MMP-1 expression was significantly reduced in 60% of EtOH extraction (Figure 2(a)). However, MMP-1 expression and collagen expression should be correlated with each other for protecting the skin. As in Figure 2(b), collagen expression was dramatically increased in 20% of EtOH extraction. Therefore, 20% of EtOH extraction was the suitable extraction condition for the antiaging of skin.

**Figure 3:**
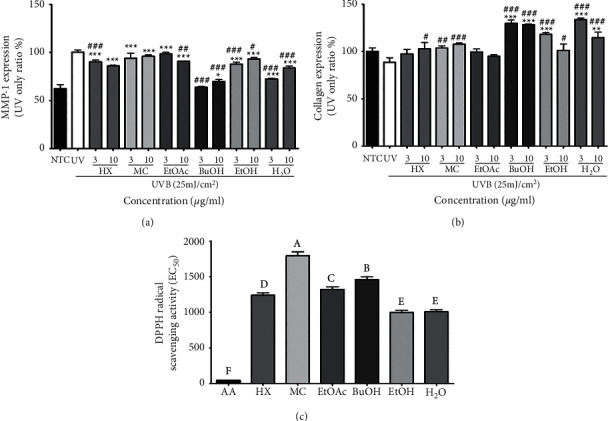
MMP-1 (a) and collagen (b) expression levels as well as DPPH radical scavenging activity (c) in HDF cells after treatment with oat fractions. Each bar represents the mean ± SD of three replicates. ^*∗*^*p* < 0.05, ^*∗∗*^*p* < 0.01, ^∗∗∗^*p* < 0.001 versus NTC group, and ^#^*p* < 0.05, ^##^*p* < 0.01, ^###^*p* < 0.001 versus UV group. Data were analyzed statistically using one-way ANOVA followed by Tukey's post hoc test. NTC: negative control; UV: ultraviolet ray irrigated group; HX: n-hexane; MC: dichloromethane; EtOAc: ethyl acetate; BuOH: *n-*butanol; EtOH: 20% ethanol extract; H_2_O: residue from fractionation.

MMP-1 expression increased by UV irradiation was reduced by all oat extracts under all fractionation conditions (Figure 3(a)). In particular, butanol at 3 *μ*g/mL downregulated MMP-1 expression to a level similar to that in the negative control group. This level was even lower than that in the group treated with water fraction. Dichloromethane (MC) and ethyl acetate (EtOAc) fractions did not clearly show an inhibitory effect on MMP-1 expression. There was no distinct pattern in MMP-1 expression according to the concentration of extract used for treatment. Collagen expression levels in groups treated with oat extract fractions at both 3 *μ*g/mL and 10 *μ*g/mL were increased compared to those in the UV-only group (Figure 3(a)). At a lower concentration of 3 *μ*g/mL, collagen expression levels after treatment with different fractions are listed in the following descending order: water, *n*-butanol, ethanol, dichloromethane (MC), ethyl acetate (EtOAc), and *n*-hexane. However, at a higher concentration of 10 *μ*g/mL, collagen expression levels were all lower than those treated at 3 *μ*g/mL except for *n*-butanol or dichloromethane. Also, DPPH radical scavenging activities of different oat fractions have been examined in HDF Cells. Antioxidant activities of different fractions are shown in Figure 3(c). Substances showing the highest radical scavenging activities were EtOH and H_2_O fractions. The dichloromethane fraction had the lowest antioxidant activity. From the result of fractionation, some functional substances were expected to be eluted with hexane. Substances showing the highest antioxidant activity were thought to be polar substances soluble in ethanol and water.

### 3.3. Activity of Oat Extract after Microbial Fermentation

**Figure 4:**
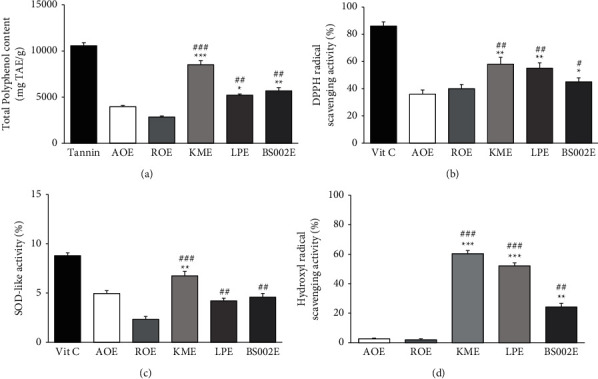
Physiological activity of fermented oat extract. (a) Total polyphenol content, (b) DPPH radical scavenging activity, (c) SOD activity, and (d) hydroxyl radical scavenging activity. Each bar represents the mean ± SD of three replicates. ^*∗*^*p* < 0.05, ^*∗∗*^*p* < 0.01, ^∗∗∗^*p* < 0.001 versus AOE group, and ^#^*p* < 0.05, ^##^*p* < 0.01, ^###^*p* < 0.001 versus ROE group. Data were analyzed statistically using one-way ANOVA followed by Tukey's post hoc test. ROE: raw oat extracted with 20% ethanol; AOE: autoclaved oat extracted with 20% ethanol; LPE: 20% ethanol extract of oats fermented by *Lactobacillus plantarum* YS-100; KME: 20% ethanol extract of oats fermented by *Kluyveromyces marxianus* YS-091.

Total phenolic content, DPPH radical scavenging activity, and SOD activity were measured to determine the antioxidant activities of fermented oats according to the type of microorganism used for fermentation (Figures 4(a)∼4(c)). Ethanol (20%) extracts of oats fermented with *Bacillus subtilis* NDJ-002 (KCCM12379P, BS002), *L. plantarum* YS-100 (KCCM12615P, LP100), and *K. marixanus* YS-091 (KCCM12635P, KM091) were used. In terms of total phenolic content, KME showed the most significant increase, higher than unfermented oat extract and autoclaved oat extract (Figure 4(a)). Since oat tissues were decomposed by microbial fermentation, the extraction efficiency of polyphenol was increased. In the case of DPPH radical scavenging activity, KME and LPE showed significantly higher antioxidant activities than controls. Increased phenolic contents of oat extracts due to fermentation might be associated with the increase of DPPH radical scavenging activity (Figure 4(b)), regarding SOD activity (Figure 4(c)), and hydroxyl radical activity (Figure 4(d)). KME showed the highest activity, confirming that KME possessed the highest overall antioxidant activity. Overall, fermentation of oats by microorganisms can increase polyphenol compounds which give rise to antioxidant activity. Among different fermentable microorganisms, KME is predicted to be able to increase antioxidant activity as it produces polyphenols by most bacteria.

### 3.4. Chemical Characteristics of Fermented Oat Extracts by GC/MS Analysis

In GC/MS analysis performed to measure volatile compounds in fermented oat extract, 10 substances were found in common (Table 3 and Supplementary Figure 1). Among these, phenolic compounds such as vanillin, 2, 4-di-tert-butylphenol, and 4-vinylphenol were detected. Organic acids such as butanoic acid, hexadecenoic acid, and hexanoic acid were also identified. 4-Vinylphenol was prepared by thermal decarboxylation of hydroxycinnamic acid from avenanthramide [29]. It is mainly used as a flavoring agent [30]. It possesses antioxidant activity. Vanillin, one representative functional substance of oats, gives oats a vanilla flavor. Studies have shown that vanillin has activities such as angiogenesis inhibiting, analgesic effect inhibiting, and antioxidant activities [29, 30]. 2, 4-Di-tert-butylphenol is a toxic phenolic compound with antioxidant, anti-inflammatory, and antibacterial activities.

### 3.5. Chemical Characteristics of Fermented Oat Extracts by LC/MS Analysis

**Figure 5:**
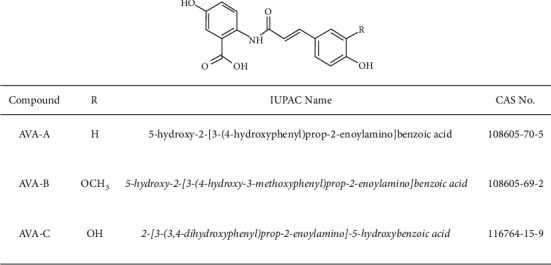
Structure of avenanthramides (AVA).

**Figure 6:**
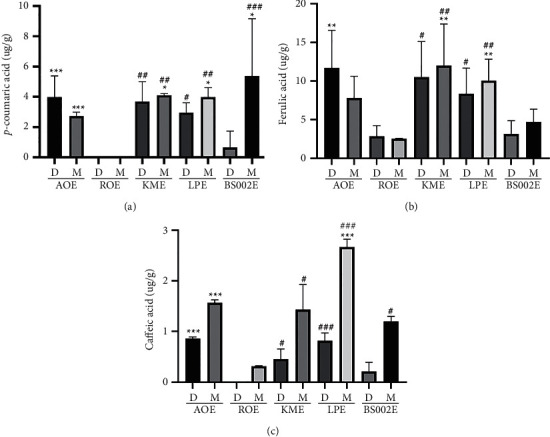
Analysis of p-coumaric acid (a), ferulic acid (b), and caffeic acid (c) as N-cinnamoyl anthranilic acid components in fermented oat extracts. Each bar represents the mean ± SD of three replicates. ^*∗*^*p* < 0.05, ^*∗∗*^*p* < 0.01, ^∗∗∗^*p* < 0.001 versus AOE group, and ^#^*p* < 0.05, ^##^*p* < 0.01, ^###^*p* < 0.001 versus ROE group. Data were analyzed statistically with one-way ANOVA followed by Tukey's post hoc test using GraphPad Prism 8.02. ROE: 20% ethanol extract of raw oat; AOE: 20% ethanol extract of autoclaved oat; LPE: 20% ethanol extract of oats fermented by *Lactobacillus plantarum* YS-100; KME: 20% ethanol extract of oats fermented by *Kluyveromyces marxianus* YS-091; BS002E: 20% ethanol extract of oats fermented by *Bacillus subtilis* NDJ-002; D extract using distilled water as a solvent; M extract using methanol as a solvent.

**Figure 7:**
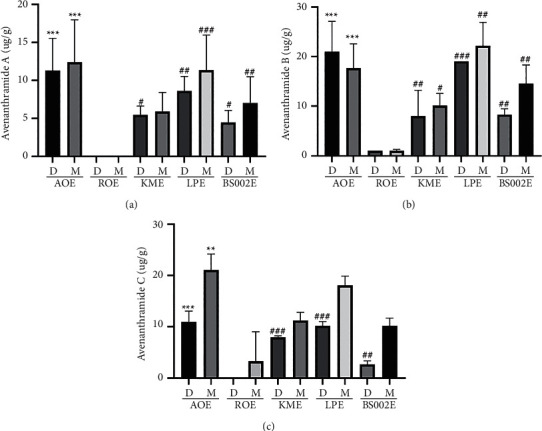
Contents of AVA-A (a), AVA-B (b), and AVA-C (c) in fermented oats. Each bar represents the mean ± SD of three replicates. ^*∗*^*p* < 0.05, ^*∗∗*^*p* < 0.01, ^∗∗∗^*p* < 0.001 versus AOE group, and ^#^*p* < 0.05, ^##^*p* < 0.01, ^###^*p* < 0.001 versus ROE group. Data were analyzed statistically with one-way ANOVA followed by Tukey's post hoc test using GraphPad Prism 8.02. ROE: 20% ethanol extract of raw oat; AOE: 20% ethanol extract of autoclaved oat; LPE: 20% ethanol extract of oats fermented by *Lactobacillus plantarum* YS-100; KME: 20% ethanol extract of oats fermented by *Kluyveromyces marxianus* YS-091; BS002E: 20% ethanol extract of oats fermented by *Bacillus subtilis* NDJ-002; D extract using distilled water as a solvent; M extract using methanol as a solvent.

Polyphenol compounds include protocatechuic acid, syringic acid, vanillin, hydroxybenzoic acid, gallic acid, coumaric acid, ferulic acid, ferulic acid, and caffeic acid have been found in oats [31]. Among them, avenanthramide, a polyphenol present in oats, has been reported to have antioxidative, anti-inflammatory, anticancer, and anti-inflammatory effects [13]. Avenanthramide is derived from the condensation of anthranilic acid and cinnamic acid linked by an amide bond (Figure 5). The cinnamic acid of AVA-A, AVA-B, and AVA-C, which are representative forms of avenanthramide, is composed of p-coumaric acid, ferulic acid, and caffeic acid, respectively. Quantification of each substance allows us to determine whether avenanthramide is degraded by microbial fermentation. Contents of p-coumaric acid and caffeic acid in water solvent of BS002E were measured to be very low (Figures 6(a), 6(b), and 6(c)). Contents of avenanthramides A, B, and C were all measured to be very small in ROE. Contents of cinnamoyl acid were also measured to be very small in ROE (Supplementary Figures 2 and 3). In the case of ROE, it could be inferred that, unlike the process of high-temperature treatment for sterilization used for other experimental groups, a heating condition up to 70°C and sufficient conditions for extracting avenanthramides A, B, and C were not provided. Avenanthramide was measured to be mostly higher in AOE where microbial fermentation was not performed than in the three conditions in which microbial fermentation was performed (Figure 7(a), 7(b), and 7(c)). In the analysis with the same solvent, all AVAs were the highest in LPE. In the case of fermented products, a larger amount was measured when dissolved in methanol than in water. A similar tendency for contents of avenanthramide and cinnamic acid has been reported by Meydani et al. [32]. When a change in AVA content during the sterilization process was confirmed, some threshed oats were lost, although AVA content was not reduced in nonthreshed oats.

Oats ingested as whole grains contain dietary fiber in the hull and bran with *β*-glucan in endosperm cell walls. Dietary fiber and *β*-glucan are considered the most representative functional nutrients of oats. As a result, it is effective food for lowering cholesterol level, reducing the risk of cardiovascular disease, and alleviating colorectal cancer [33, 34]. In the case of cereals, biochemical properties can be improved by the fermentation process with microorganisms. In particular, functionalities appear by modifying phenolic compounds such as carotenoid, inulin, and *β*-glucan. However, the substrate of microorganisms is limited to starch in refined grains [24]. In this experiment, three types of microorganisms typically used for fermentation were selected. Changes in functionality and expression levels of functional substances were then confirmed. *L. plantarum* is a Gram-positive, bacilli-shaped bacterium. *L. plantarum* mainly produces lactic acid, although it also produces acetic acid, ethanol, and carbon dioxide [35]. *L. plantarum* is industrially used to make silage or ferment food. In the food industry, it is mainly used for the fermentation of protein foods such as cheese, sausage, and fish, although it is also used to ferment vegetables such as kimchi and pickles [36]. Among dietary fibers, *β*-glucan, in particular, controls postprandial blood glucose and increases the transport and excretion of bile acids by adjusting the absorption rate in the digestive tract through its characteristic of high viscosity.

When stimulated by UV rays, ROS-mediated DNA damage and apoptosis in human skin epidermal cells lead to skin aging. The process of removing ROS before it triggers an intracellular reaction is thus important to alleviate skin aging [37]. Vitamin E, phenolic compounds, phytic acid, flavonoids, and avenanthramides are antioxidant components of oats [38]. Avenanthramide has a structure in which anthranilic acid and hydrocinnamic acid are bonded by a peptide bond. Several studies have demonstrated that avenanthramide of oats is a representative antioxidant active substance [13]. In order to use the benefit of fermented oat extract, it is necessary to isolate it in an ester-bonded state from the cell wall [30]. Bratt et al. [39] have reported that its antioxidant activity is different depending on the type of cinnamic acid, a constituent compound of avenanthramide, showing the following activity order: (AVA)-C > AVA-B > AVA-A. The difference in antioxidant activity is due to a functional group where caffeic acid and ferulic acid having one hydrogen atom at the *R* position have higher nonenzymatic oxidation than coumaric acid having two hydrogen atoms [40]. During enzymatic oxidation, dehydrodicinnamic acid dilactone is formed by oxidoreductase due to a coupling reaction of phenoxyl radical and an intramolecular nucleophilic attack of carboxylic acid moieties [41]. However, depending on the solvent, the hydrogen atom removal process is more affected by properties between the antioxidant and the solvent than by the type of radical. This reaction is affected by the hydrogen bond between -OH and -NH contained in the antioxidant and the solvent [42].

In Wu's study, the amount of phenolic compound, hydroxyl radical scavenging, and peroxide anion scavenging activity in extracts of wheat germ inoculated with *L. plantarum* tended to increase as the fermentation process proceeded [43]. In the present study, the results of total phenolic contents were inconsistent with DPPH radical scavenging, similar to the results of Wu's study. Thus, the antioxidant activity might be due to both polyphenolic compounds and soluble proteins decomposed by microorganisms [44].

The fermentation process can increase the production of peptides due to protein hydrolysis with the separation and dissolution of phenolic compounds contained in grain tissue [45, 46]. *K. marxianus* is an aerobic yeast in the family of *Saccharomyces*, the oldest microbial yeast species used for fermentation purposes. It is a representative microorganism that converts sugars into alcohol through respiratory fermentation metabolism [47]. *K. marxianus* can produce saccharides and inulin to exert its advantages such as thermotolerance and high growth ability compared to similar yeast *Saccharomyces cerevisiae* and *K. lactis* [48]. Industrially, *K. marxianus* is used to produce biomass [49] or ethanol from whey or lactose [50]. It is used for the production of endogenous enzymes such as *β*-xylosidase, *β*-glucosidase, and inulinase [51] as well as ingredients widely used in the food industry such as emulsifier mannoprotein [52] and baker's yeast [53]. Studies related to antioxidant activity have revealed that carbohydrates with low molecular weight show high antioxidant activity due to the degradation of saccharides by yeast [54]. A protein hydrolysate of *K. marxianus* shows cellular antioxidant activity [55].

The polarity of a compound is an intrinsic property, which is determined by the size of the substance, the number and position of functional groups, and electronegativity. The active ingredient is thus identified through separation. Purification can increase the purity of a substance. Physicochemical properties can be assessed by an extraction method using the polarity of substances. Substances with a small difference in polarity between the solvent and the active fraction can be separated. In this experiment, an ethanol extract having a weak polarity was fractionated by utilizing low to high polar solvents as follows: hexane having a polarity of 0, dichloromethane having a polarity of 3.1, ethyl acetate having a polarity of 4.4, butanol having a polarity of 4, and water having a polarity of 9. Thus, the active ingredient was separated according to the polarity of the substance. Among well-known active ingredients of oats, organic acids such as ferulic acid and caffeic acid are considered to possess high polarity whereas dietary fiber and *β*-glucan are expected to be eluted from solvents with a low polarity. Substances with a high polarity are typically organic acids. *β*-glucan is partially soluble in water and a solvent with a high polarity. Due to its physical property, it plays an important role in the absorption of other nutrients and functional substances. It is also important for the delivery to the digestive system [56].

In this study, functional substances of fermented oat extracts and inhibitory effects against skin aging induced by ultraviolet rays of fermented oat extracts were determined. According to results from MMP-1 and collagen expression assays in HDF cells, 20% ethanol extract with fractionation was the most suitable solvent for extraction. When the extract was fractionated, the butanol fraction showed the best skin-protecting effect. When the antioxidant activity of the fermented extract was measured, KME showed the highest activity. Quantification of AVA using LC/MS showed that AVA content was higher in the nonfermented AOE than in microbially fermented oat extracts.

## 4. Conclusions

Fermented with YS-091 oat extracts treatment showed inhibitory effects of antiaging on the skin with functional substances than nonfermented oat extracts.

## Figures and Tables

**Table 1:** LC/MS analysis conditions.

Item	Conditions		
Column	BEH 2.1 (2.7 mm × 50 mm, 1.8 *μ*m)		
Column TeM	40		

Mobile phase	A: 0.1% folic acid (FA)		
	B: Acetonitrile (ACN)		

Flow rate	0.5 mL/min		
Injection volume	3 *μ*L		

Gradient	Min	A	B
	0	95	5
	0.5	95	5
	1	50	50
	1.5	50	50
	2	5	95
	3	5	95
	5	95	5

ESI + capillary	2KV		
Source TeM desolvation TeM	250		
Source gas flow (L/Hr)	600		
Source temp	150		

Avenanthramide A: ES^+^, chan reaction: 300.18 > 147.11, cone voltage: 32, col. energy: 10, avenanthramide B: ES^+^, chan reaction: 316.30 > 163.11, cone voltage: 22, col. energy: 10, avenanthramide C: ES^+^, chan reaction: 330.19 > 177.12, cone voltage: 42, col. energy: 12, p-coumaric acid: ES^−^, chan reaction: 163.10 > 119.10, cone voltage: 40, col. energy: 13, caffeic acid: ES^−^, chan reaction: 179.10 > 135.10, cone voltage: 35, col. energy: 15, ferulic acid: ES^−^, chan reaction: 193.10 > 134.10, cone voltage: 40, col. energy: 15.

**Table 2:** Extract yield and DPPH radical scavenging activity of the oat according to various ethanol concentrations.

Extract condition (EtOH %)	Yield (%)	DPPH (EC50, *μ*g/mL)
0	12.5	1,715.4 ± 152.2
20	18.9	951.9 ± 77.2
40	22.2	1,631.0 ± 131.2
60	22.8	1,213.7 ± 122.8
70	23.2	732.6 ± 67.7
80	20.1	489.9 ± 45.8
100	1.2	290.6 ± 24.2
Ascorbic acid		3.0

**Table 3:** Major compounds of the oat extracted by 20% ethanol confirmed by GC/MS.

No	Major compounds	Homology (%)				
		ROE	AOE	BS002E	LPE	KME
1	Vanillin	88	87	89	94	90
2	2,4-Di-tert-butylphenol	87	95	97	97	97
3	Benzene,1-3-bis (1,1-dimethylethyl)	78	85	94	94	88
4	4-Vinylphenol	43	70	82	78	71
5	2,3-Butanediol	78	80	83	83	88
6	Butanoic acid	70	71	89	74	85
7	Benzeneacetaldehyde	64	78	80	92	89
8	n-Formyl-beta-alanine	28	29	49	54	56
9	Hexadecanoic acid	88	77	79	97	97
10	Hexanoic acid	75	76	77	77	80

ROE: raw oat 20% ethanol extract, AOE: autoclaved oat 20% ethanol extract, BS002E: 20% ethanol extract that fermented by *Bacillus subtilis* NDJ-002, LPE: 20% ethanol extract that fermented by *Lactobacillus plantarum* YS-100, KME: 20% ethanol extract that fermented by *Klyuverpmyces marixanus* YS-091.

## Data Availability

The data that support the findings of this study are available from the corresponding author on reasonable request.

## Supplementary Materials

Supplementary Materials Supplementary Figure 1. GC-MSanalysis of AOE, BSO2E, KME, LPE, and ROE. Supplementary Figure 2. Quantification of avenanthramides A, B, and C. (A. Standard curve. (B) AOE, ROE, BSOOE, KME, and LPE. Supplementary Figure 3. Quantification of coumaric acid, caffeic acid, ferulic acid, and vanillin quantification. (A) Standard curve. (B) AOE, ROE, BSOOE, KME, and LPE.
