# Supplementary material for: Bacterially Converted Oat Active Ingredients Enhances Antioxidative and Anti-UVB Photoaging Activities
Source: Evid Based Complement Alternat Med. 2022 May 28;2022:1901564. doi: 10.1155/2022/1901564 (PMC9187468; doi:10.1155/2022/1901564)

Supplementary Figure 1. GC-MS analysis of AOE, BSO2E, KME, LPE and ROE

AOE

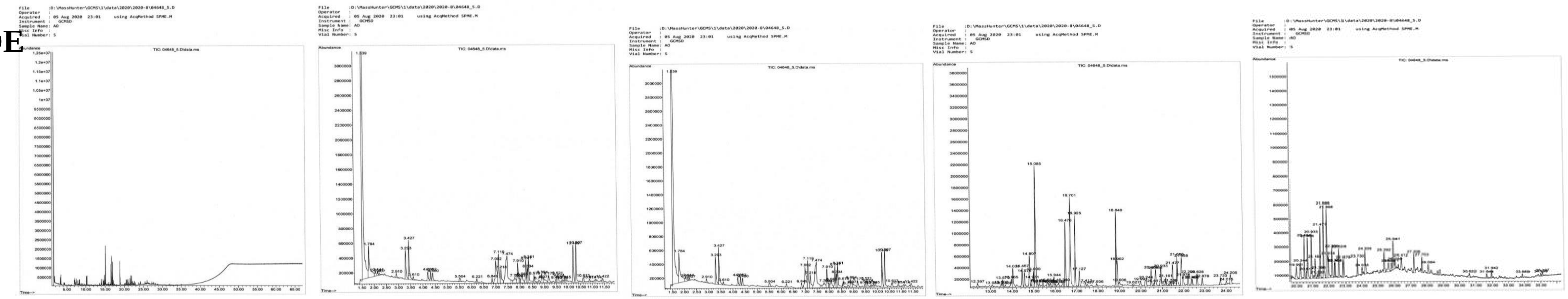

BSO2E

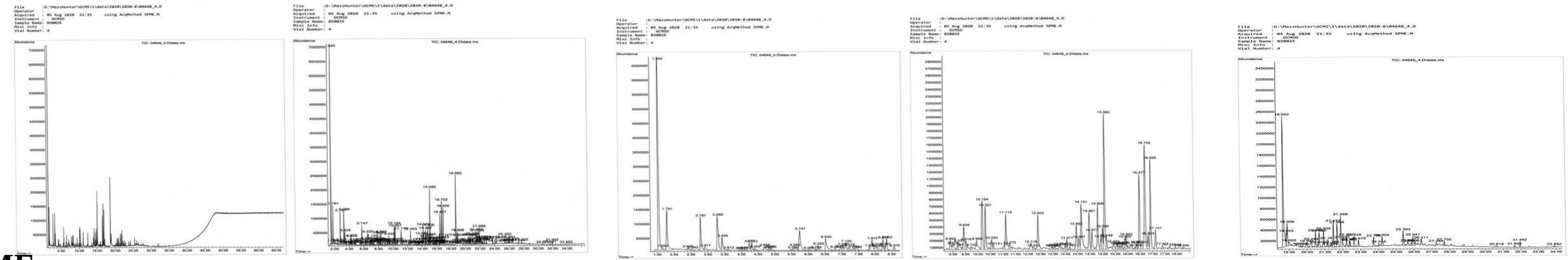

KME

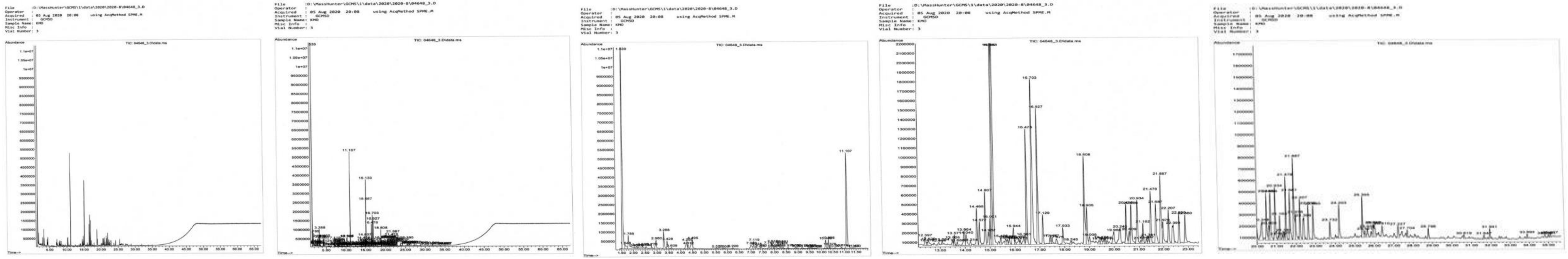

LPE

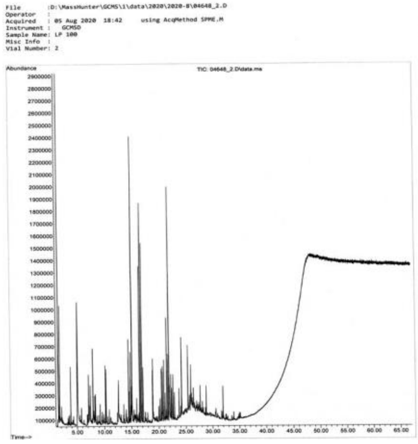
File :D:\MassHunter\GCMS\1\data\2020\2020-8\04648_2.D
Acquired : 05 Aug 2020 18:42 using AcqMethod SPME.M
Instrument : GCMSD
Sample Name: LP 100
Vial Number: 2
TIC: 04648_2.D\data.ms

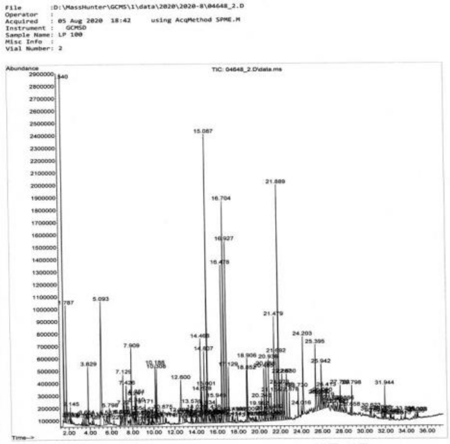
File :D:\MassHunter\GCMS\1\data\2020\2020-8\04648_2.D
Acquired : 05 Aug 2020 18:42 using AcqMethod SPME.M
Instrument : GCMSD
Sample Name: LP 100
Vial Number: 2
TIC: 04648_2.D\data.ms

ROE

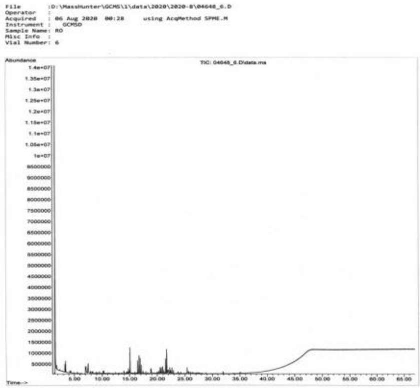
File :D:\MassHunter\GCMS\1\data\2020\2020-8\04648_6.D
Acquired : 06 Aug 2020 00:28 using AcqMethod SPME.M
Instrument : GCMSD
Sample Name: RO
Vial Number: 6
TIC: 04648_6.D\data.ms

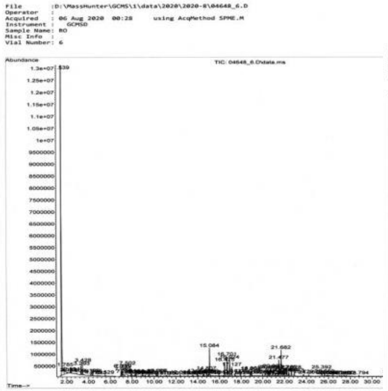
File :D:\MassHunter\GCMS\1\data\2020\2020-8\04648_6.D
Acquired : 06 Aug 2020 00:28 using AcqMethod SPME.M
Instrument : GCMSD
Sample Name: RO
Vial Number: 6
TIC: 04648_6.D\data.ms

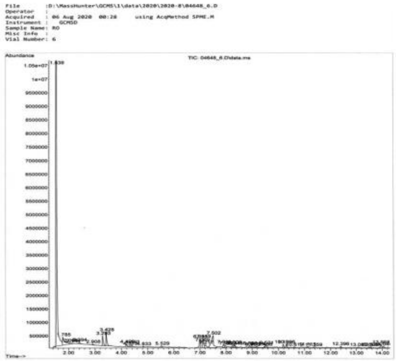
File :D:\MassHunter\GCMS\1\data\2020\2020-8\04648_6.D
Acquired : 06 Aug 2020 00:28 using AcqMethod SPME.M
Instrument : GCMSD
Sample Name: RO
Vial Number: 6
TIC: 04648_6.D\data.ms

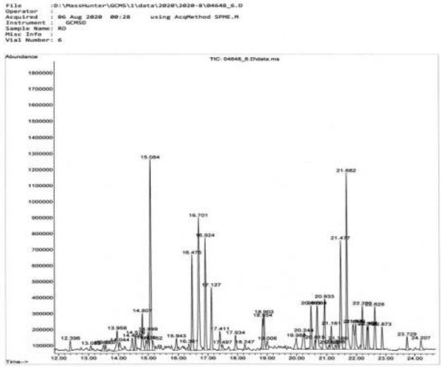
File :D:\MassHunter\GCMS\1\data\2020\2020-8\04648_6.D
Acquired : 06 Aug 2020 00:28 using AcqMethod SPME.M
Instrument : GCMSD
Sample Name: RO
Vial Number: 6
TIC: 04648_6.D\data.ms

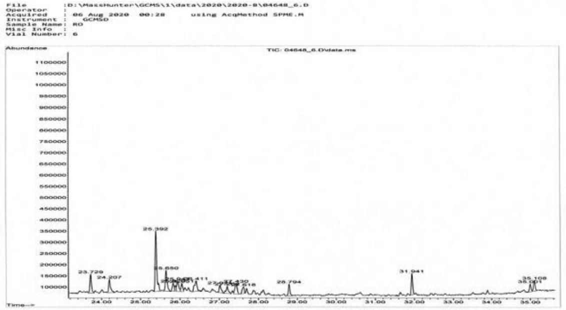
File :D:\MassHunter\GCMS\1\data\2020\2020-8\04648_6.D
Acquired : 06 Aug 2020 00:28 using AcqMethod SPME.M
Instrument : GCMSD
Sample Name: RO
Vial Number: 6
TIC: 04648_6.D\data.ms

Supplementary Figure 2. Quantification of avenanthramides A, B and C. A) Standard curve. B) AOE, ROE, BSOOE, KME and LPE

A)

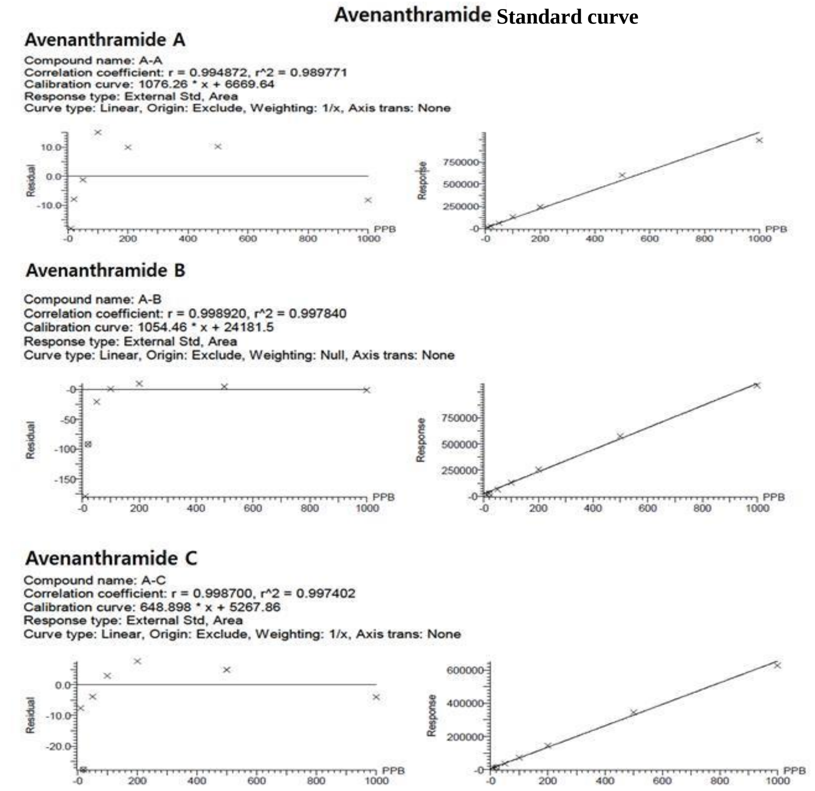

B)

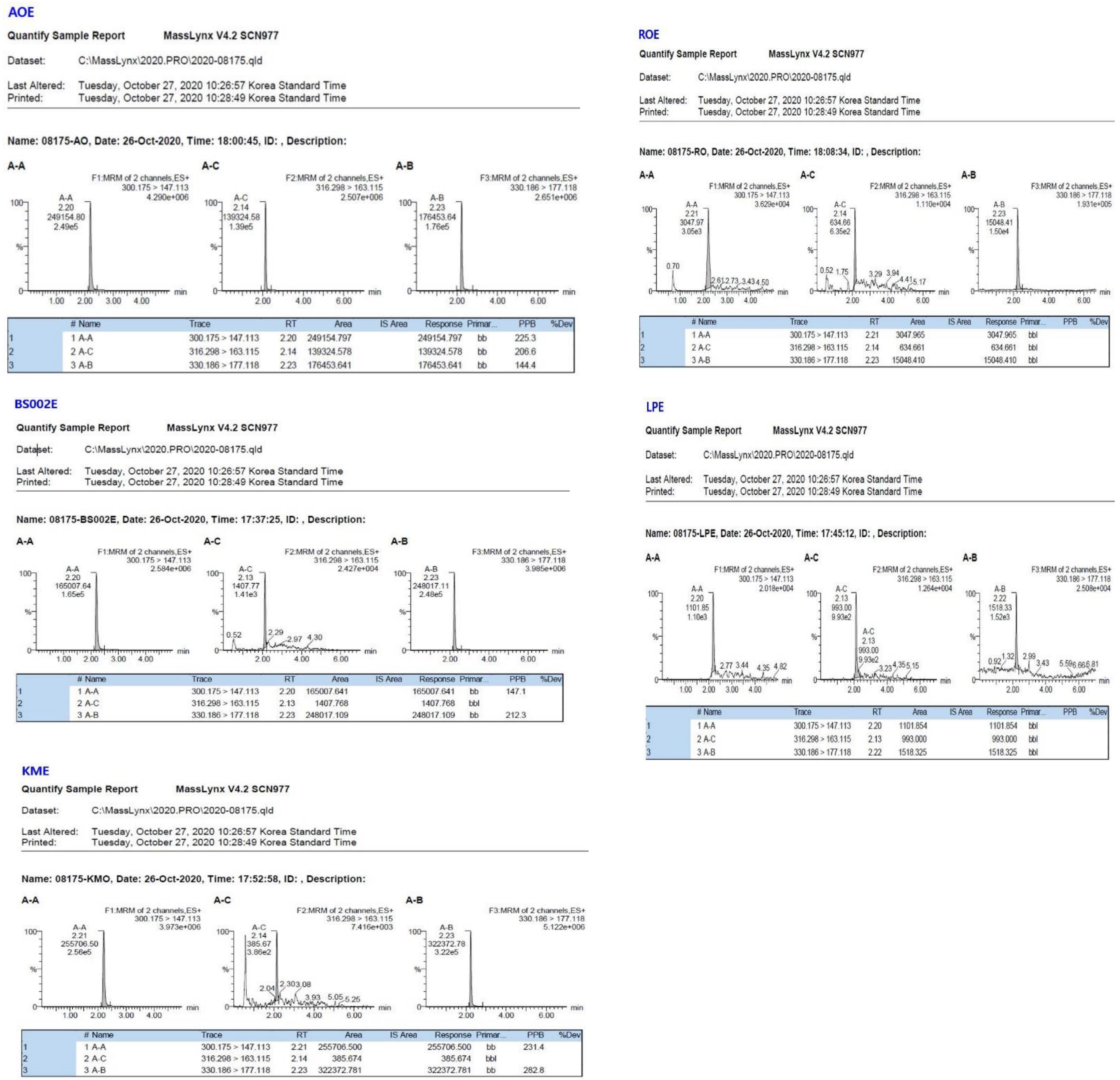

AOE

Quantify Sample Report MassLynx V4.2 SCN977

Dataset: C:\MassLynx\2020.PRO\2020-08175.qld

Last Altered: Tuesday, October 27, 2020 10:26:57 Korea Standard Time
Printed: Tuesday, October 27, 2020 10:28:49 Korea Standard Time

Name: 08175-AO, Date: 26-Oct-2020, Time: 18:00:45, ID: , Description:

	# Name	Trace	RT	Area	IS Area	Response	Primar...	PPB	%Dev
1	1 A-A	300.175 > 147.113	2.20	249154.797		249154.797	bb	225.3	
2	2 A-C	316.298 > 163.115	2.14	139324.578		139324.578	bb	206.6	
3	3 A-B	330.186 > 177.118	2.23	176453.641		176453.641	bb	144.4	

ROE

Quantify Sample Report MassLynx V4.2 SCN977

Dataset: C:\MassLynx\2020.PRO\2020-08175.qld

Last Altered: Tuesday, October 27, 2020 10:26:57 Korea Standard Time
Printed: Tuesday, October 27, 2020 10:28:49 Korea Standard Time

Name: 08175-RO, Date: 26-Oct-2020, Time: 18:08:34, ID: , Description:

	# Name	Trace	RT	Area	IS Area	Response	Primar...	PPB	%Dev
1	1 A-A	300.175 > 147.113	2.21	3047.965		3047.965	bbl		
2	2 A-C	316.298 > 163.115	2.14	634.661		634.661	bbl		
3	3 A-B	330.186 > 177.118	2.23	15048.410		15048.410	bbl		

BS002E

Quantify Sample Report MassLynx V4.2 SCN977

Dataset: C:\MassLynx\2020.PRO\2020-08175.qld

Last Altered: Tuesday, October 27, 2020 10:26:57 Korea Standard Time
Printed: Tuesday, October 27, 2020 10:28:49 Korea Standard Time

Name: 08175-BS002E, Date: 26-Oct-2020, Time: 17:37:25, ID: , Description:

	# Name	Trace	RT	Area	IS Area	Response	Primar...	PPB	%Dev
1	1 A-A	300.175 > 147.113	2.20	165007.641		165007.641	bb	147.1	
2	2 A-C	316.298 > 163.115	2.13	1407.768		1407.768	bbl		
3	3 A-B	330.186 > 177.118	2.23	248017.109		248017.109	bb	212.3	

LPE

Quantify Sample Report MassLynx V4.2 SCN977

Dataset: C:\MassLynx\2020.PRO\2020-08175.qld

Last Altered: Tuesday, October 27, 2020 10:26:57 Korea Standard Time
Printed: Tuesday, October 27, 2020 10:28:49 Korea Standard Time

Name: 08175-LPE, Date: 26-Oct-2020, Time: 17:45:12, ID: , Description:

	# Name	Trace	RT	Area	IS Area	Response	Primar...	PPB	%Dev
1	1 A-A	300.175 > 147.113	2.20	1101.854		1101.854	bbl		
2	2 A-C	316.298 > 163.115	2.13	993.000		993.000	bbl		
3	3 A-B	330.186 > 177.118	2.22	1518.325		1518.325	bbl		

KME

Quantify Sample Report MassLynx V4.2 SCN977

Dataset: C:\MassLynx\2020.PRO\2020-08175.qld

Last Altered: Tuesday, October 27, 2020 10:26:57 Korea Standard Time
Printed: Tuesday, October 27, 2020 10:28:49 Korea Standard Time

Name: 08175-KMO, Date: 26-Oct-2020, Time: 17:52:58, ID: , Description:

	# Name	Trace	RT	Area	IS Area	Response	Primar...	PPB	%Dev
1	1 A-A	300.175 > 147.113	2.21	255706.500		255706.500	bb	231.4	
2	2 A-C	316.298 > 163.115	2.14	385.674		385.674	bbl		
3	3 A-B	330.186 > 177.118	2.23	322372.781		322372.781	bb	282.8	

Supplementary Figure 3. Quantification of coumacic acid, caffeic acid, ferulic acid and canillin quatification. A) Standard curve. B) AOE, ROE, BSOOE, KME and LPE

A)

Standard curve for courmaric acid

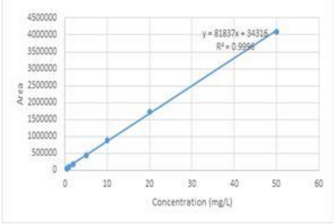

Standard curve for caffeic acid

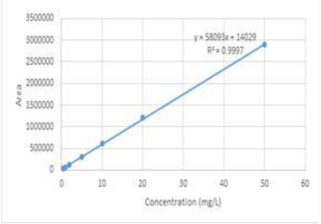

Standard curve for ferulic acid

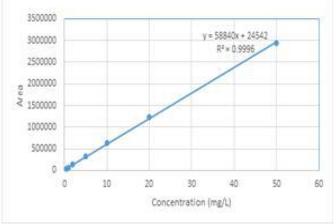

Standard curve for vanillin

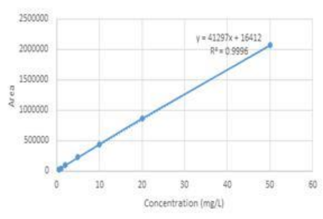

B)

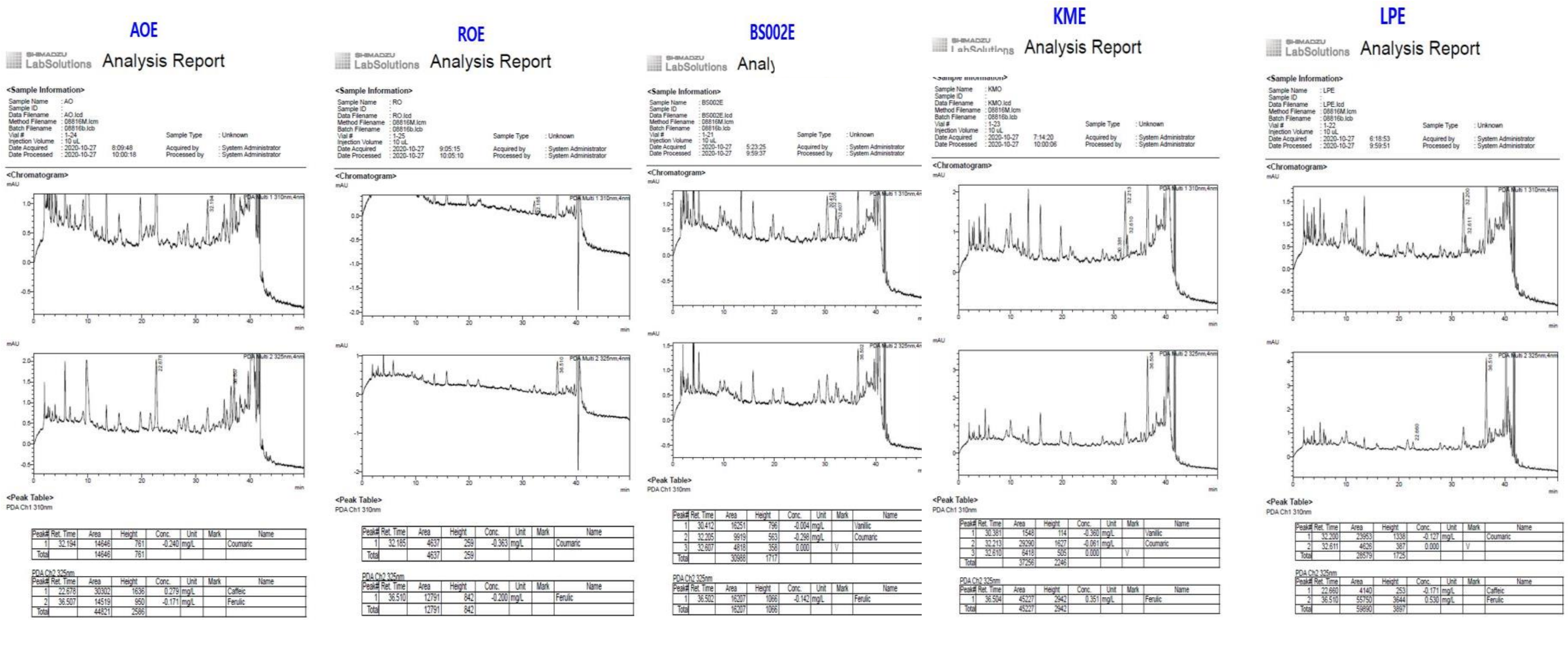

Supplement: Supplementary Materials — Supplementary Figure 1. GC-MSanalysis of AOE, BSO2E, KME, LPE, and ROE. Supplementary Figure 2. Quantification of avenanthramides A, B, and C. (A. Standard curve. (B) AOE, ROE, BSOOE, KME, and LPE. Supplementary Figure 3. Quantification of coumaric acid, caffeic acid, ferulic acid, and vanillin quantification. (A) Standard curve. (B) AOE, ROE, BSOOE, KME, and LPE. [file 1901564.f1.pdf]
